# Preoperative Magnetic Seed Versus Wire-Guided Localization in the Treatment of Nonpalpable Breast Cancer: A Retrospective Observational Study at a Tertiary Care Center

**DOI:** 10.1155/ijbc/9960666

**Published:** 2025-09-12

**Authors:** Amedeo Villanucci, Francesca Romana Ferranti, Sonia Cappelli, Flavia Cavicchi, Franco Graziano, Federica Lauria, Fabio Pelle, Ilaria Puccica, Isabella Sperduti, Antonello Vidiri, Claudio Botti

**Affiliations:** ^1^Department of Breast Surgery, IRCCS Regina Elena National Cancer Institute, Rome, Italy; ^2^Department of Radiology, IRCCS Regina Elena National Cancer Institute, Rome, Italy; ^3^Department of Surgical Sciences, Sapienza University of Rome, Rome, Italy; ^4^Biostatistics, IRCCS Regina Elena National Cancer Institute, Rome, Italy

**Keywords:** breast cancer, magnetic seed, magseed, nonpalpable, wire-guided

## Abstract

**Background:** Breast cancer screening and effective neoadjuvant treatments have increased surgeries for nonpalpable tumors, often requiring preoperative localization. The wire-guided method, performed on the same day as surgery, has limitations, prompting interest in wire-free alternatives like magnetic seed devices.

**Methods:** A retrospective single-center study (November 2020–March 2024) compared magnetic seed and wire-guided localization in 558 patients. The primary aim was to assess localization and retrieval success, resection margins, and reoperation rates. Secondary endpoints included the interval between localization and surgery, operative time, incision site selection, and volume excised.

**Results:** Among 558 patients, 188 underwent magnetic seed and 370 wire-guided localizations. Both groups were similar in BMI, breast size, and lesion characteristics. Complications in the wire-guided group included device migration (0.5%) and hematoma (1.3%). Success rates were comparable (98.9% vs. 99.7%), as were positive margins (5.3% vs. 6.7%) and reoperation rates (6.9% vs. 7.8%). Excised volume was significantly lower in the magnetic seed group (24.2 [range 6.5–48.0 cm^3^] vs. 41.5 cm^3^ [range 16.0–68.0 cm^3^], *p* < 0.001). The magnetic seed group had an average localization-to-surgery interval of 1 day (range 0–160 days).

**Conclusions:** Magnetic seed localization is as safe and effective as wire-guided localization, with comparable success rates and resection margins adequacy. Its primary advantage is scheduling flexibility, offering a longer interval between localization and surgery.

## 1. Introduction

In recent decades, the implementation of mammographic screening programs, along with improved imaging technologies, has led to a significant increase in the diagnosis of breast cancer at a very early stage [1]. Usually, these lesions are not clinically evident and may present with different radiological features, including small nodular opacities, architectural distortion, and microcalcifications. In most cases, patients are candidates for breast-conserving surgery (BCS), provided that the target lesion is accurately localized preoperatively to allow the surgeon to perform a complete cancer clearance with adequate resection margins while preserving as much healthy tissue as possible. Similarly, the increased use of primary systemic therapy for neoadjuvant purposes (neoadjuvant systemic therapy [NAST]), even in early stage breast cancer, has expanded the indication for BCS due to its high effectiveness in tumor downsizing [2]. The response of the cancer to the preoperative treatment also raises the issue of preoperative localization of the target lesion, as it is no longer clinically evident at the time of surgery. The method commonly used for the localization of such lesions is the placement of a wire (wire guided [WG]) under ultrasound or mammographic guidance, which currently represents the standard of care due to extensive clinical experience and supporting long-term data [3]. Common limitations of wires include the risk of dislocation or migration, and difficulties in surgical scheduling, since the wire must be inserted on the same day as the surgery. Moreover, the location for wire insertion is usually selected based on the radiologist's ease, without consideration of the surgeon's preference for the skin incision site. Due to the distance between the wire entry point on the breast surface, the target lesion, and the incision site, the surgeon might be forced to perform extensive breast tissue dissection and overresection [4]. Alternative wire-free techniques have been described over time in the literature to overcome some limitations of the WG method, including techniques using radionuclides, intraoperative ultrasound, radiofrequency devices, or radar reflectors [5–9]. Among these, the use of the paramagnetic device Magseed (Endomag, Cambridge, United Kingdom) has gained popularity since its introduction [10]. Magseed (MS) is a 5 × 1 mm cylindrical device that can be inserted through a needle under local anesthesia into the target lesion with ultrasound or mammographic guidance and subsequently identified intraoperatively with a dedicated probe magnetometer (Sentimag, Endomag, Cambridge, United Kingdom). Although the literature on this subject has grown considerably in recent years [11–17], there are relatively few comparative studies between the conventional method and the use of MS [18–22]. The aim of this study was to contribute to the evidence currently available on the topic by retrospectively comparing the cases of nonpalpable breast cancer treated in our institute with the MS method to those treated with the WG method during the same period.

## 2. Materials and Methods

### 2.1. Patients' Selection and Study Design

We conducted a single-center retrospective study involving patients diagnosed with nonpalpable breast cancer who were candidates for BCS and required preoperative localization of the target lesion. These patients underwent treatment at the Breast Surgery Department of the National Cancer Institute “Regina Elena” of Rome from November 2020 to March 2024. After selecting the patients, we divided the study population into two cohorts based on whether they had undergone the MS or WG procedure. Since its introduction in November 2020 at our institution, the use of MS has gradually increased, and by the time the study closed, both methods were equally used. The inclusion criteria for this study were as follows: age ≥ 18 years, breast cancer histologically confirmed by previous ultrasound-guided core-needle biopsy (CNB) or stereotactic vacuum-assisted biopsy (VABB), and nonpalpable lesion requiring preoperative localization, including patients with significant clinical response after primary systemic therapy for neoadjuvant purposes, regardless of nodal status at the time of surgery. To make the study population as homogeneous as possible, we excluded all cases where the procedure was performed for complex multifocal lesions that required the placement of more than one wire or MS, lesions classified as B3, and lesions not previously characterized by CNB or VABB. The localization procedure was performed under ultrasound or mammographic guidance, depending on the radiological features of the target lesion. Radiograms of the surgical specimen were not routinely performed in both groups but were done as needed to confirm the adequacy of breast resection, especially in cases involving microcalcifications or patients treated with neoadjuvant therapy. Data were extracted from patients' hospital records, breast radiology registries, surgical logs, and histopathology reports. Specifically, the following data were collected and analyzed: sociodemographic and morphometric data, including age, body mass index, and breast size; clinical data, including radiological presentation, size and depth of the lesion, histotype, rate of positive margins, and rate of reintervention; and technical data, including localization and retrieval success rate, interval from localization to surgery, duration of surgery, incision site, and volume excised. The primary endpoint of our study was to evaluate the effectiveness of the MS method compared with WG in terms of success rates for the localization and removal of the target lesion. Secondary endpoints were to compare oncological safety of the two methods regarding the rate of positive margins and reintervention (excluding reconstruction or remodeling procedures) and evaluate the impact of the MS method on the duration of surgery, the choice of the incision site, and the excised volume.

### 2.2. Statistical Analysis

Given the retrospective nature of the study, the baseline characteristics of patients treated with the two methods may have been unbalanced. For this reason, the propensity score matching technique was used to create two groups of patients with similar probabilities of assignment to treatment in order to achieve a comparison with reduced selection bias. Descriptive statistics were calculated for all variables of interest. Categorical variables were reported as absolute frequencies and relative percentage values, while continuous variables were summarized using medians and ranges. All associations between categorical variables were evaluated by Pearson's chi-square test or Fisher's exact test. For quantitative variables, the differences between the two methods were evaluated with the nonparametric Mann–Whitney test or the parametric Student's *t*-test. The choice of parametric or nonparametric test was made after the normality of the data distribution was verified by the Shapiro–Wilk test. In all cases, *p* values ≤ 0.05 were considered statistically significant. All analyses were carried out using SPSS V29.0 (IBM Corporation, Armonk, New York, United States).

## 3. Results

### 3.1. Population Characteristics

We included a total of 558 patients: 188 in the MS group and 370 in the WG group. Sociodemographic and morphometric data are shown in Table 1. The median age was lower in the WG group than in the MS group, although they did not differ significantly in terms of BMI or breast size. Radiological features of cancer are summarized in Table 1, with nodular opacities and glandular distortions being more prevalent in the MS group and microcalcifications in the WG group. No significant differences were found regarding the depth or size of the lesions. Cases treated with neoadjuvant therapy were significantly higher in the WG group (4.8% vs. 14.9%, *p* < 0.0001). The pathological findings showed a higher prevalence of invasive carcinoma in the MS group and carcinoma in situ in the WG group, reflecting the higher prevalence of microcalcifications in the latter. Similarly, the complete response rate was higher in the WG group, consistent with the higher prevalence of neoadjuvant therapy in this cohort, although no difference was found in terms of receptor status between the two groups. Furthermore, no differences were found between the two groups regarding histotype (ductal NST vs. lobular vs. other special type).

### 3.2. Oncological and Technical Endpoints

The success rate of the procedure, defined as the accuracy of radiological localization and lesion retrieval during surgery, did not show significant differences between the two groups, with a success rate of 98.9% in the MS group and 99.7% in the WG group. In both groups, only one case of accurate localization was not followed by successful lesion retrieval, as confirmed by the final histopathological report. Additionally, we found one case of a failed localization procedure in the MS group, attributed to the extremely small size of the target lesion. Patients in the WG group underwent preoperative localization on the same day as their planned surgery, in accordance with device safety protocols. In contrast, patients in the MS group underwent the procedure up to 160 days before surgery, with a median 1-day interval between localization and surgery. No complications related to the localization procedure were encountered in the MS group; however, a 1.3% complication rate was observed in the WG group, including two cases of wire migration and three cases of intramammary hematoma. These results are summarized in Table 2. The rate of positive resection margins did not show significant differences between the two groups (5.3% vs. 6.7%), nor did the rate of reintervention for oncological reasons (6.9% vs. 7.8%). Similarly, no differences were found in the type of reintervention between the two groups. Finally, the analysis of data on the technical aspects of the surgical procedures revealed no significant differences between the two groups in terms of operative time, the choice of incision site, or the associated type of axillary surgery. However, excised volume was significantly lower in the magnetic seed group (24.2 [range 6.5–48.0 cm^3^] vs. 41.5 cm^3^ [range 16.0–68.0 cm^3^], *p* < 0.001). When considering the excised volume, all cases of oncoplastic procedures were not included in the analysis as the amount of tissue removed is intentionally greater and therefore could have biased the comparison.

## 4. Discussion

WG localization for nonpalpable breast cancer remains the most commonly used technology among breast centers in Italy [23]. However, it has several limitations, including potential misalignment between the insertion and ideal incision sites, the risk of wire displacement or breakage, time constraints between placement and surgery, and patient discomfort due to the protruding wire. The MS system was introduced in 2016 as an alternative wire-free method for nonpalpable breast lesion localization, and the first study to assess its oncological safety and feasibility appeared in the literature in 2018 [10]. In this initial study, the authors reported accurate localization, no migration, and intraoperative detectability in a wide range of breast sizes and at all depths in a small patient cohort undergoing total mastectomy. Since then, evidence supporting the MS system's applicability and efficacy for its intended purpose in BCS has progressively increased. As experience with this technique has grown over time, the indications for MS localization have been further extended to include marking metastatic axillary lymph nodes or other nonpalpable malignancies of soft tissues [24–28]. The present study is aimed at assessing the effectiveness of the MS method compared to WG for the successful localization and removal of target lesions, while also comparing oncological safety, reintervention rates, and the impact on the choice of incision site. To our knowledge, only a few studies directly compare the two methods [18–22]. Based on our analysis, both MS and WG localization techniques demonstrated similarly high success rates for the accurate localization and removal of breast cancer lesions. The MS group achieved a 98.9% success rate, while the WG group slightly outperformed it, with a success rate of 99.7%. These high success rates align with the existing literature on the MS system. No significant differences were observed between the two groups in terms of oncological safety, as measured by the rates of positive resection margins (5.3% in MS vs. 6.7% in WG) and reintervention rates (6.9% in MS vs. 7.8% in WG). These findings suggest that both MS and WG techniques offer comparable oncological efficacy in achieving tumor-free margins during BCS. The higher rate of neoadjuvant therapy in the WG group (14.9% vs. 4.8% in MS) reflects variations in patient selection but did not lead to significant differences in clinical outcomes. This bias could be related to the use of breast magnetic resonance imaging (MRI) in disease restaging after NAST. Although the MS device is certified for conditional MRI compatibility, artifacts in the image generation could compromise the accuracy of the evaluation. Recently, a study demonstrated the reliability of excising residual breast cancer lesions following NAST using long-term MS localization, followed by contrast-enhanced spectral mammography (CESM) to evaluate response [29]. This study showed that MS did not affect the accuracy of response evaluation according to RECIST criteria. Given the potential utility of MS as a disease marker in patients undergoing NAST, it is desirable to increase the use of CESM for restaging and to develop further clinical feasibility studies. In terms of technical aspects, our study showed that the MS technique offers a distinct advantage over the WG method in terms of scheduling flexibility. MS localization can be performed days before surgery, with a median interval of 1 day (range: 0–160 days). Conversely, WG localization must be performed on the same day as the surgery. This scheduling flexibility could greatly facilitate surgical planning and patient management, reducing the need for same-day coordination between radiology and surgery. Moreover, MS was not associated with a longer surgery duration, regardless of the type of associated axillary procedure. Based on our experience, the MS system involved a minimal learning curve for both radiologists and surgeons, with proficiency typically achieved after the first few procedures. Intraoperative detection was consistently reliable, though occasional signal attenuation occurred in cases involving large breast size or deeply positioned seeds; these instances were manageable with proper probe positioning and calibration. Notably, we encountered no cases of seed migration, aligning with existing literature on the device's stability. As noted earlier, one of the criticisms of WG localization is its limitation on the surgeon's choice of incision site, which is influenced by the wire's insertion point. Achieving a satisfactory cosmetic outcome is a key objective of BCS, and certain incision patterns, including periareolar, inframammary fold, or oncoplastic approaches, can aid in achieving this goal. Since the use of MS theoretically allows the surgeon more flexibility in choosing the incision site, while still adhering to the tumor's location, we expected a significant difference in incision site selection between the two groups. Interestingly, our findings showed that MS did not significantly impact the surgeon's ability to select the optimal incision site. This may be attributed to advances in surgical planning or the expertise of surgeons at our tertiary center, indicating that outcomes between the two methods are likely equivalent when performed by skilled practitioners. Notably, our study found that the excised volume was significantly lower in the MS group compared to the WG localization group (24.2 [range 6.5–48.0 cm^3^] vs. 41.5 cm^3^ [range 16.0–68.0 cm^3^], *p* < 0.001). The significantly lower excised volume observed in the MS group may be explained by the enhanced intraoperative precision provided by the Sentimag probe. Unlike wire localization, which provides a fixed linear directional guide, the MS system enables real-time, multidirectional detection of the seed with auditory and numeric feedback. This allows the surgeon to localize the lesion three-dimensionally, minimizing unnecessary removal of healthy tissue and preserving breast esthetics without compromising oncological safety, even when the skin incision pattern remains similar. Several limitations should be considered when interpreting the results of this study. As a retrospective study, there is an inherent risk of bias, although we attempted to mitigate this by applying propensity score matching. In our study, patient assignment to localization method was not randomized but based on logistical availability and clinical judgment during the study period, particularly as MS was gradually introduced into routine practice. Nevertheless, we observed no differences in breast size, lesion size, or lesion depth between the MS and WG groups, suggesting that MS was feasible and effective across diverse anatomical scenarios. These findings support the notion that MS does not have specific indications and could, in principle, fully replace WG localization for nonpalpable breast lesions, considering its demonstrated benefits. The main limitation to its broader adoption remains its higher cost, particularly in healthcare systems with constrained budgets. Although in this study a formal cost analysis was not conducted, it is important to consider the economic implications of MS localization compared to WG, especially since cost is a significant factor in healthcare decision-making. While MS localization has higher upfront costs, WG is a well-established, low-cost method that has been the standard of care for decades. However, the increased scheduling flexibility offered by MS could offset these costs in the long term, particularly in high-turnover settings where logistical efficiency is a priority. Further limitations of the study include the relatively short follow-up period limited to immediate surgical outcomes and the lack of patient-reported outcomes. Moreover, the study's outcomes might be influenced by the high level of surgical expertise at our tertiary center, which may not reflect the outcomes in centers with less experienced surgeons and fewer patients.

## 5. Conclusions

This retrospective study comparing MS and WG localization techniques for nonpalpable breast cancer showed similarly high success rates for both methods in lesion localization and removal. Oncological safety was comparable between the two methods, with similar rates of positive margins and reinterventions. The MS technique offered a distinct advantage in scheduling flexibility, as the seed could be placed days in advance of surgery, enhancing patient and resource management. Contrary to expectations, MS did not significantly impact the surgeon's choice of incision site, suggesting that both methods are equally effective when performed by skilled surgeons. Although MS has higher upfront costs, its scheduling benefits may improve logistical efficiency. Further studies, including cost-effectiveness analyses are needed to fully assess its clinical and economic value.

## Figures and Tables

**Table 1:** Sociodemographic and morphometric data, clinical and radiological features, and histopathological characteristics.

	**MS**	**WG**	**p** ** value**
Patients included	188	370	
Age	65 (37–90) years	61 (29–88) years	0.003^∗^
BMI	25.2 (18.1–45.9) kg/m^2^	24.9 (18.0–49.5) kg/m^2^	0.503
Breast cup size			0.166
Small (up to 300 cm^3^)	46 (24.5%)	120 (32.4%)	
Medium (300–600 cm^3^)	89 (47.3%)	161 (43.5%)	
Large (600–1200 cm^3^)	51 (27.1%)	88 (23.8%)	
Gigantomastia (over 1200 cm^3^)	2 (1.1%)	1 (0.3%)	
Radiological features			*p* < 0.0001^∗^
Nodular opacity	157 (83.5%)	289 (78.1%)	
Distorsion	25 (13.3%)	19 (5.1%)	
Microcalcifications	6 (3.2%)	39 (10.5%)	
Others	0 (0.0%)	23 (6.2%)	
Size of lesion	10 (1–25) mm	10 (2–35) mm	0.335
Depth	1.30 (0.41–2.88) cm	1.38 (0.35–6.40) cm	0.065
Neoadjuvant	9 (4.8%)	55 (14.9%)	*p* < 0.0001^∗^
Pathology findings			*p* < 0.0001^∗^
Invasive	122 (64.9%)	193 (52.2%)	
In situ	6 (3.2%)	37 (10.0%)	
Invasive + in situ	57 (30.3%)	108 (29.2%)	
Other	1 (0.5%)	1 (0.3%)	
CPR	2 (1.1%)	31 (8.4%)	
Histotype			0.335
Ductal NST	155 (82.4%)	294 (87.0%)	
Lobular	24 (12.8%)	30 (8.9%)	
Other special type	9 (4.8%)	14 (4.1%)	
Receptor status^a^			0.748
ER/PR + HER2−	3 (33.3%)	14 (25.5%)	
ER/PR + HER2+	4 (44.4%)	22 (40.0%)	
ER/PR–HER2+	0 (0.0%)	6 (10.9%)	
TN	2 (22.2%)	13 (23.6%)	

Abbreviations: BMI, body mass index; CPR, complete pathological response; ER, estrogen receptor; HER2, human epidermal growth factor Receptor 2; NST, no special type; PR, progesterone receptor; TN, triple negative.

^a^Data refer to patients who underwent neoadjuvant therapy.

∗*p* < 0.05 were considered statistically significant.

**Table 2:** Primary endpoint, oncological endpoint, and operative data.

	**MS**	**WG**	**p** ** value**
Procedure outcome			0.331
Lesion localized and retrieved	186 (98.9%)	369 (99.7%)	
Lesion localized and NOT retrieved	1 (0.5%)	1 (0.3%)	
Lesion NOT localized	1 (0.5%)	0 (0.0%)	
Localization-to-surgery interval	1 (0–160) day	0 day	
Complications	0 (0%)	5 (1.3%)	0.278
Migration	0 (0%)	2 (0.5%)	
Hematoma	0 (0%)	3 (0.8%)	
Positive margins	10 (5.3%)	25 (6.7%)	0.503
Reintervention	13 (6.9%)	29 (7.8%)	0.684
Type of reintervention			0.116
Widening	5 (2.7%)	7 (1.9%)	
Mastectomy	5 (2.7%)	21 (5.7%)	
SLNB	3 (1.6%)	1 (0.3%)	
ALND	0 (0.0%)	3 (0.8%)	
Pattern of skin incision			0.086
Radial/lazy S	74 (39.4%)	126 (34.0%)	
Curved/semicircular	20 (10.6%)	21 (5.7%)	
Periareolar	57 (30.3%)	145 (39.2%)	
IMF	27 (14.4%)	54 (14.6%)	
Oncoplastic^a^	10 (5.3%)	24 (6.5%)	
Duration of surgery	60 (10–185) min	55 (22–192) min	0.268
Axillary surgery associated			0.291
SLNB	160 (85.1%)	327 (88.4%)	
ALND	16 (8.5%)	26 (7.8%)	
TAD	3 (1.6%)	9 (2.4%)	
None	9 (4.8%)	8 (2.2%)	
Excised volume^b^	24.2 (6.5–48.0) cm^3^	41.5 (16.0–68.0) cm^3^	*p* < 0.001^∗^

Abbreviations: ALND, axillary lymph node dissection; IMF, inframammary fold; SLNB, sentinel lymph node biopsy; TAD, targeted axillary dissection.

^a^Including Benelli, Batwing, J-plasty, and Wise patterns.

^b^Excluding patients who underwent oncoplastic procedures.

∗*p* < 0.05 were considered statistically significant.

## Data Availability

Due to patient privacy and ethical restrictions, the data underlying this study cannot be made publicly available. However, anonymized data may be obtained from the corresponding author upon reasonable request and subject to institutional approval.

## References

[1] Verdial F. C., Etzioni R., Duggan C., Anderson B. O. (2017). Demographic Changes in Breast Cancer Incidence, Stage at Diagnosis and Age Associated With Population-Based Mammographic Screening. *Journal of Surgical Oncology*.

[2] Early Breast Cancer Trialists’ Collaborative Group (EBCTCG) (2018). Long-Term Outcomes for Neoadjuvant Versus Adjuvant Chemotherapy in Early Breast Cancer: Meta-Analysis of Individual Patient Data From Ten Randomised Trials. *The Lancet Oncology*.

[3] Chan B. K., Wiseberg-Firtell J. A., Jois R. H., Jensen K., Audisio R. A., Cochrane Breast Cancer Group (2016). Localization Techniques for Guided Surgical Excision of Non-Palpable Breast Lesions. *Cochrane Database of Systematic Reviews*.

[4] Cheung B. H. H., Co M., Lui T. T. N., Kwong A. (2024). Evolution of Localization Methods for Non-Palpable Breast Lesions: A Literature Review From a Translational Medicine Perspective. *Translational Breast Cancer Research*.

[5] Paganelli G., Veronesi U. (2002). Innovation in Early Breast Cancer Surgery: Radio-Guided Occult Lesion Localization and Sentinel Node Biopsy. *Nuclear Medicine Communications*.

[6] Gray R. J., Salud C., Nguyen K. (2001). Randomized Prospective Evaluation of a Novel Technique for Biopsy or Lumpectomy of Nonpalpable Breast Lesions: Radioactive Seed Versus Wire Localization. *Annals of Surgical Oncology*.

[7] Haid A., Knauer M., Dunzinger S. (2007). Intra-Operative Sonography: A Valuable Aid During Breast-Conserving Surgery for Occult Breast Cancer. *Annals of Surgical Oncology*.

[8] Lowes S., Bell A., Milligan R., Amonkar S., Leaver A. (2020). Use of Hologic LOCalizer Radiofrequency Identification (RFID) Tags to Localise Impalpable Breast Lesions and Axillary Nodes: Experience of the First 150 Cases in a UK Breast Unit. *Clinical Radiology*.

[9] Mango V. L., Wynn R. T., Feldman S. (2017). Beyond Wires and Seeds: Reflector-Guided Breast Lesion Localization and Excision. *Radiology*.

[10] Price E. R., Khoury A. L., Esserman L. J., Joe B. N., Alvarado M. D. (2018). Initial Clinical Experience With an Inducible Magnetic Seed System for Preoperative Breast Lesion Localization. *American Journal of Roentgenology*.

[11] Harvey J. R., Lim Y., Murphy J. (2018). Safety and Feasibility of Breast Lesion Localization Using Magnetic Seeds (Magseed): A Multi-Centre, Open-Label Cohort Study. *Breast Cancer Research and Treatment*.

[12] Murphy E., Quinn E., Stokes M. (2022). Initial Experience of Magnetic Seed Localization for Impalpable Breast Lesion Excision: First 100 Cases Performed in a Single Irish Tertiary Referral Centre. *Surgeon*.

[13] Powell M., Gate T., Kalake O., Ranjith C., Pennick M. O. (2021). Magnetic Seed Localization (Magseed) for Excision of Impalpable Breast Lesions-The North Wales Experience. *The Breast Journal*.

[14] Liang D. H., Black D., Yi M. (2022). Clinical Outcomes Using Magnetic Seeds as a Non-Wire, Non-Radioactive Alternative for Localization of Non-Palpable Breast Lesions. *Annals of Surgical Oncology*.

[15] Crèvecoeur J., Jossa V., Di Bella J., Coibion M., Crèvecoeur A. (2023). Clinical Experience of the Magseed Magnetic Marker to Localize Non-Palpable Breast Lesions: A Cohort Study of 100 Consecutive Cases. *Gland Surgery*.

[16] Constantinidis F., Sakellariou S., Chang S. L. (2022). Wireless Localisation of Breast Lesions With Magseed. A Radiological Perspective of 300 Cases. *The British Journal of Radiology*.

[17] Depretto C., Della Pepa G., De Berardinis C. (2023). Magnetic Localization of Breast Lesions: A Large-Scale European Evaluation in a National Cancer Institute. *Clinical Breast Cancer*.

[18] Zacharioudakis K., Down S., Bholah Z. (2019). Is the Future Magnetic? Magseed Localisation for Non Palpable Breast Cancer. A Multi-Centre Non Randomised Control Study. *European Journal of Surgical Oncology*.

[19] Micha A. E., Sinnett V., Downey K. (2021). Patient and Clinician Satisfaction and Clinical Outcomes of Magseed Compared With Wire-Guided Localisation for Impalpable Breast Lesions. *Breast Cancer*.

[20] Redfern R. E., Shermis R. B. (2022). Initial Experience Using Magseed for Breast Lesion Localization Compared With Wire-Guided Localization: Analysis of Volume and Margin Clearance Rates. *Annals of Surgical Oncology*.

[21] Shirazi S., Hajiesmaeili H., Khosla M., Taj S., Sircar T., Vidya R. (2023). Comparison of Wire and Non-Wire Localisation Techniques in Breast Cancer Surgery: A Review of the Literature With Pooled Analysis. *Medicina*.

[22] Moreno-Palacios E., Martí C., Frías L. (2024). Breast-Conserving Surgery Guided With Magnetic Seeds vs. Wires: A Single-Institution Experience. *Cancers*.

[23] Giuliani G., Vitale R., Brunetti N. (2024). Non-Palpable Breast Lesions Localization Techniques - A New Priority: Results of a Senonetwork Survey Among Breast Centers in Italy. *Annals of Surgical Oncology*.

[24] Mariscal Martínez A., Vives Roselló I., Salazar Gómez A. (2021). Advantages of Preoperative Localization and Surgical Resection of Metastatic Axillary Lymph Nodes Using Magnetic Seeds After Neoadjuvant Chemotherapy in Breast Cancer. *Surgical Oncology*.

[25] Reitsamer R., Peintinger F., Forsthuber E., Sir A. (2021). The Applicability of Magseed for Targeted Axillary Dissection in Breast Cancer Patients Treated With Neoadjuvant Chemotherapy. *Breast*.

[26] Martínez M., Jiménez S., Guzmán F., Fernández M., Arizaga E., Sanz C. (2022). Evaluation of Axillary Lymph Node Marking With Magseed Before and After Neoadjuvant Systemic Therapy in Breast Cancer Patients: MAGNET Study. *The Breast Journal*.

[27] van der Burg S. J. C., Kuijpers A., Baetens T. (2024). Magnetic Seed Localization Is Feasible for Non-Palpable Melanoma, Merkel Cell Carcinoma, and Soft Tissue Sarcoma Lesions. *European Journal of Surgical Oncology*.

[28] Shabo I., Zouzos A., Fredholm H. (2024). Magseed Application for Detecting Recurrent Lymph Node Metastasis in Papillary Thyroid Cancer: A Novel Minimally Invasive Approach. *European Journal of Surgical Oncology*.

[29] Bravo E. I., Martínez A. M., Alvà H. P. (2024). Reliability of Magseed Marking Before Neoadjuvant Systemic Therapy With Subsequent Contrast-Enhanced Mammography in Patients With Non-Palpable Breast Cancer Lesions After Treatment: The MAGMA Study. *Breast Cancer Research and Treatment*.

